# Could MicroRNA be Neurological Prognosis Biomarkers after Cardiac Arrest?

**DOI:** 10.4274/TJAR.2024.241557

**Published:** 2024-05-03

**Authors:** Şule Özbilgin, Necati Gökmen

**Affiliations:** 1 Dokuz Eylül University Faculty of Medicine, Department of Anaesthesiology and Intensive Care, İzmir, Turkey

**Keywords:** Biomarker, cardiac arrest, intensive care, microRNAs, neurological function, prognostics

## Abstract

For patients monitored in intensive care units in the aftermath of a cardiac arrest, one of the well-established difficulties of care after resuscitation is the ability to perform the necessary prognostic assessments as accurately and early as possible. Although current guidelines include algorithms to determine prognosis, there are still missing links and uncertainties. Biomarkers obtained from peripheral blood are generally non-invasive and easy to obtain. Although the potential to use microRNA as a prognostic biomarker after cardiac arrest has received less interest recently, its popularity has increased in the last few years. By identifying prognostic biomarkers within 24 h of cardiac arrest, clinicians in intensive care could gain valuable insights to guide patient outcomes and predict both mortality and survival rates.

**Main Points**

• Pinpointing a patient’s prognosis after successful resuscitation remains a murky picture, largely clouded by the heavy use of medications such as sedatives, opioids, and neuromuscular blockers.

• The quest to accurately assess brain damage and forecast neurological recovery requires innovative approaches.

• MicroRNA accurately predicts good and poor neurological outcomes.

• MicroRNA is an accurate and early marker of long-term neurological outcomes following cardiac arrest.

## Introduction

A leading contributor to both mortality and morbidity, sudden cardiac arrest claims the lives of over a million individuals worldwide each year.^1^ Approximately 300,000 cardiac arrests occur in Europe annually, with 85% of these cases ending in death.^2^ Early prediction of neurological outcomes following successful cardiopulmonary resuscitation using simple clinical examination is challenging. The introduction of therapeutic hypothermia as a treatment for comatose cardiac arrest patients has added another layer of complexity to the already challenging task. The pharmacokinetic profile of sedative agents is impacted by hypothermia, and effects that prolong sedation have been identified as a major factor in the decreased predictive power of clinical neurological examination in predicting unfavorable outcomes following cardiac arrest.^3,4^

Therefore, new techniques are required to quantify the extent of brain damage and forecast the results. Numerous circulatory biomarkers, including neurofilament light chain protein levels, neuron-specific enolase (NSE), and S100B, were evaluated in relation to prognosis. The effectiveness of these evaluations varied, and while resuscitation science guidelines stipulate that they should inform prognostic forecasts, the hunt for the perfect biomarker continues.^5,6,7^ Prognostic assessment following cardiac arrest is now performed using a combination of neurological examination, biochemical and neurophysiological testing, and imaging a few days post-event.^1,6^

The primary challenge in predicting the early prognosis of individuals who are resuscitated after cardiac arrest is the absence of precise biomarkers.^8^ As mentioned above the most reliable measures of neurological outcomes were, until recently, neurophysiological testing and clinical neurological examination performed a few days after arrest.^9,10^ Guidelines and clinical practice propose using biomarkers in circulation, such as NSE, because they improve outcome prediction determination.^11,12^ Nevertheless, novel biomarkers must be used for the therapy management of individual patients, and the discriminatory power of these neurophysiological tests needs to be enhanced for each patient individually.

## What is the Role of microRNAs?

### In Prognosis

MicroRNAs (miRNAs) discovery in 2001 sparked intense attention in the scientific community.^13,14,15^ Their potential significance as novel disease biomarkers was highlighted by their stability and presence in blood circulation.^16,17^ MicroRNAs in circulation has been used as a biomarker of cardiovascular disorders in several studies. While research has primarily focused on heart attacks and failing hearts, the potential of miRNAs as a prognostic tool during cardiac arrest has only recently begun to shine a light on its possible role.^8^

Short RNA molecules (around 21 nucleotides) known as miRNAs do not encode for proteins. These are often expressed molecules, despite being somewhat tissue specific and having been maintained by evolution. Since the MicroRBase Sequence Database’s debut in December 2002,^8^ the catalog of known miRNAs has been steadily blossoming. In 2578 individuals, up to 30,424 miRNAs of 206 kinds have been identified.^8^ Despite being unable to code for proteins, miRNAs play crucial roles in regulating their genes and, consequently, the expression of proteins. By causing mRNA degradation, miRNA predominantly controls animal gene expression.^18^ This characteristic guarantees that miRNA can control physiological, pathological and developmental processes.^19^

Numerous processes in the heart, including apoptosis, angiogenesis, contraction, and hypertrophy are regulated by miRNAs.^20^ Similarly, miRNAs are highly expressed in the brain.^21^ These characteristics make them essential for developing the brain and diseases.^22^

The results of PubMed screening of 624 papers suggested that miRNA might be a possible biomarker in neurological disease, according to a systematic review by Devaux et al.^8^ Much of the research has focused on the usefulness of miRNA as a brain tumor diagnostic biomarker. Seven hundred twenty-four articles have discussed miRNAs as cardiovascular disease biomarkers. The majority of them were found to concentrate on heart failure (106 articles) and myocardial infarcts (125 articles).

There is a shortage of papers of research findings that solely consider cardiac arrest, and most of these studies seem to evaluate the diagnostic rather than prognostic potential of miRNA.

In one study, miRNAs levels were examined in the first 48 and 24 h following return of spontaneous circulation (ROSC) in 65 patients who had therapeutic hypothermia following cardiac arrest. Six months after cardiac arrest, miRNA levels were assessed using the cerebral performance category (CPC) score, which was divided into two categories; good neurological outcome (CPC score 1 or 2) and poor neurological outcome (CPC score 3-5). After 48 h, there were no discernible variations in the levels of miR-146a, miR-122, miR-208b, miR-21, miR-9, and miR-128 between the groups with favorable and poor neurological outcomes. On the other hand, in the 24 h and 48 h following cardiac arrest, miR-124 was sharply higher in patients who had positive outcomes than in those who had bad outcomes.^1^ The researchers concluded that miR-124 was a groundbreaking new biomarker for predicting the prognosis of the nervous system in the post-cardiac arrest period based on these data. They concluded that miRNA might be a crucial factor in determining neurological outcomes and death following cardiac arrest on the basis of the data they had collected.^1^

### In Cardiac Diseases

MicroRNAs has been suggested in many studies as a potential diagnostic biomarker for cardiovascular disorders, notably acute myocardial infarcts.^8^ A major challenge in cardiovascular research is identifying valid biomarkers frequently measurable in readily available samples such as plasma. MicroRNAs have been studied for their potential as biomarkers for cardiovascular disorders because of their stability in circulation.^23^ Currently, many models of circulating miRNAs have been discovered for heart failure, atherosclerotic disease, hypertension, type 2 diabetes, and myocardial infarctions.^23^ Other studies^24,25,26,27,28^ have examined the predictive significance of circulating miRNAs following acute myocardial infarctions.

### In Brain Injury

Previous studies have examined the functions of miRNA in nervous system illnesses, including cancer, as well as in brain development and plasticity.^21,22,29,30^ The brain and other peripheral organs require oxygen and nutrients after cardiac arrest because perfusion stops. Following cerebral ischemia, the brain’s miRNA expression alterations^31^ lead to many miRNA candidates. Following ischemia/reperfusion injury, microglial-mediated neuronal death was controlled by miR-181c in a rat global cerebral ischemia model.^32^ MiRNA-181c directly targeted the area where TNF-α mRNA does not undergo 3’ translation, inhibiting apoptosis triggered by TNF-α generated in active microglial cells.^32^ This research illuminated the critical role of miRNAs as conductors of neurological dysfunction in the brain starved of oxygen. Consequently, miRNAs hold promise as both harbingers of neurological outcomes and potential targets for orchestrating neuroprotective therapies after cardiac arrest.

### In Terms of Neurological Protection

Scientists have identified a squad of miRNAs as potential warriors in the fight to shield the oxygen-deprived brain. Among them, valproic acid, a molecule known to unlock the cell’s hidden potential, emerged as a champion.^33^ In rodent stroke models, it not only mitigated neurological after effects but also revved up motor function, all while influencing the expression of miR-331 in affected brain cells.^31^ Simultaneous administration of bortezomib, a drug that tackles rogue proteins in a specific cancer myeloma,^34^ and tissue plasminogen activator, along with its neuroprotective effects in older rats after stroke, was linked to a surge in miR-146a levels within the brain’s endothelial cells.^31,35^ The million-dollar question remains can these miRNAs be individual heroes in protecting the brain, or do they require backup?

## MicroRNAs as a Prognostic Biomarker after Cardiac Arrest

Studies have proposed that specific miRNAs in blood may have value as a biomarker after cerebral ischemia due to ischemic cerebrovascular accidents occurring in both animal models^36^ and human patients.^37^ Some of these miRNAs may also be promising diagnostic tools for ischemic stroke.^37,38,39^

A study on determining the power of miRNA in predicting prognosis after cardiac arrest compared plasma miRNA in patients with favorable outcomes after cardiac arrest with patients with poor outcomes.^40^ They identified an outcome-linked miRNA biological signature using microsequences encompassing nearly 700 miRNAs (miRBAS version 12.0). Among miRNAs expressed differently in patients with positive and negative outcomes, miR-122 and miR-21 strongly predicted both death rates and neurological function after 6 months. Stammet et al.^40^ included 28 patients who survived more than 48 h after cardiac arrest. During the post-cardiac arrest period, patients underwent therapeutic hypothermia at a target core body temperature of 33 °C for 24 h. Blood samples were collected using microarray and PCR to determine miRNA expression levels 48 h after cardiac arrest in normothermic conditions using citrate tubes. Patients underwent neurological evaluations both before discharge from the intensive care unit and at the 6-month follow-up. It was determined that miR-122 and miR-21 predicted neurological outcomes and were associated with patient mortality rates. When the miRNA expression profiles in the plasma of patients after cardiac arrest were characterized, it was understood from the study results that neurological outcomes in these patients were associated with a miRNA bio-signature. The miR-122 and miR-21 levels assessed in circulation at physiological body temperature 48 h after cardiac arrest in patients treated with hypothermia were increased in patients with poor outcomes and provided some ability to forecast outcomes. Additionally, based on evidence that miR-122 and miR-21 are produced by neuronal cells, the increase in plasma concentration. In the post-cardiac arrest period was considered to be due to injured neurons. This situation motivated the assessment of how miRNA impacts our understanding of patient recovery potential in broader cardiac arrest patient cohorts.

The hypothesis that miRNAs source in neurons dying after cardiac arrest can be measured in blood circulation was confirmed by demonstrating that miR-122 and miR-21 are consistently produced by neurons, as evidenced by other studies.^41,42^ Similar to this hypothesis, another study showed that exosomes carrying miRNAs outside cells might pass the blood-brain barrier.^43^ In addition, cerebral ischemia disrupted the blood-brain barrier, which may facilitate the release of neuron-derived miRNA into blood circulation easier.^44^ The multicenter studies performed by Devaux et al.^45^, with participation from nine countries, researched the prognostic value of miRNA levels in patients with ROSC. Of the 579 patients, 304 (52.5%) had poor neurological outcomes in the sixth month (CPC scores 3 and 4). In 50 patients, brain-enriched miR-124-3p level was defined as a predictive biomarker of neurological results with short RNA sequencing, and miR-124-3p levels were significantly elevated in patients with unfavorable outcomes compared with those with good outcomes. In univariate analysis, miR-124-3p levels were strongly correlated with neurological outcomes, whereas multivariate analysis using logistic regression independently associated miR-124-3p levels with neurological outcomes. In the advanced statistical analysis models developed in this study, higher miR-124-3p levels were proven to be remarkably associated with lower survival. This evidence-based data demonstrated that miR-124-3p levels could be used as a prognostic tool for neurological outcomes and survival after non-hospital cardiac arrest. The potential of miRNA profiling to be a valuable tool for stratifying patient care following cardiac arrest.

In another study,^46^ lower miR-122-5p and higher miR-124-3p levels predicted shorter survival. The results of this new investigation showed that miR-122-5p levels measured in the 48^th^ hour after ROSC were a unique predictor influencing both brain function and survival rates and had prognostic value.

Building on the work of Sheinerman et al.^47^, researchers identified brain-specific miRNAs in the blood of patients experiencing early-stage mild cognitive decline, a hallmark of various neurodegenerative diseases. Therefore, it is understood that brain-derived miRNAs found in circulation after cardiac arrest may indicate neurological impairment. As the dimension of neurological injury emerges as a crucial factor in determining the recovery potential after cardiac arrest, miRNAs in circulation in this environment is expected to have significant prognostic value. A new study showing that brain-enriched miR-124 was associated with neurological outcomes after cardiac arrest solidly validated this initial belief.^1^ The study by Gilje et al.^1^ researched the effect on mortality and neurological prognosis by assessing plasma levels of miRNA specific to selected tissues in cardiac arrest syndrome. Previous studies primarily investigated tissue samples collected after a 48-h window following cardiac arrest. As a result, Gilje et al.^1^ assessed this time point first and assessed differences if present. They concluded that miR-124 levels were higher in the 24^th^ hour, and it may show great potential as a novel marker for use in predicting outcomes after cardiac arrest. As a result that supports clinical efficacy, it provided 97% specificity and 53% sensitivity for the prognosis of CPC score 3-5 in the 24^th^ hour. The miR-124 in plasma was previously characterized as a biomarker for brain injury in a stroke model in animal studies; however, the study by Gilje et al.^1^ is the first human study to associate miR-124 with brain injury. In this study, miR-124 was compared with NSE in prognostic terms, and no increase in diagnostic accuracy was not identified by combining these two biomarkers.

In a study to assess the correlation between miRNAs in circulation and cerebral complications following resuscitation from cardiac arrest. Stefanizzi et al.^48^ found strong correlations between three different brain-enriched miRNAs (miR9-3p, miR124-3p, and miR129-5p) and NSE. The authors stated that this result may indicate that miRNAs reflects the degree of brain injury. In addition, these miRNAs were associated with neurological outcomes and 6-month survival. These data support the importance of brain-enriched miRNA in predicting mortality and neurological outcomes after cardiac arrest. MicroR-124 was shown to be a promising new biomarker to predict prognosis for patients treated with hypothermia after cardiac arrest, according to receiver operating characteristic area under the curve, which was calculated as 0.87 in the 24^th^ hour and 0.89 in the 48^th^ hour.

Moving forward, well-designed studies are crucial to validate the potential advantages of this novel biomarker category against existing methods. In addition to the sensitivity and specificity of miRNA, their benefit for early prediction must be superior to available electrophysiological and neuroimaging tools. Finally, techniques for determining miRNA amounts are time-consuming and must be developed in terms of repeatability, speed, cost, and standardization.

Accurate assessment of prognosis shortly after cardiac arrest remains a significant hurdle. The widespread use of sedatives, pain relievers, and muscle relaxants complicates the picture. Additionally, the previously established 72-h window for neurological evaluation is no longer reliable because of the lingering effects of these medications, hindering a clear assessment of brain function. The discovery of gene expression regulators of miRNAs caused high excitement among researchers. Fueled by the potential of these molecules, numerous studies have explored their application as diagnostic tools, prognostic indicators, and therapeutic targets for cerebral and cardiovascular diseases. A critical gap exists in our knowledge regarding the use of miRNA as a biomarker for patients who have survived cardiac arrest. Future studies ideally target researching the superior value of miRNA compared with available prognostic tools and the most appropriate time to collect blood samples. While previous research has demonstrated the early release of cardiac-enriched miRNA following cardiac injury, a crucial next step lies in precisely characterizing the release pattern of brain-enriched miRNA in this context. It is essential to note the following basic technical topics related to the measurement of miRNA levels in circulation: the advantages and disadvantages of measuring miRNA in plasma compared with whole blood. Interestingly, miRNAs may be a new biomarker and potential therapeutic target after cardiac arrest.
